# Peanut skin phenolic extract attenuates hyperglycemic responses *in vivo* and *in vitro*

**DOI:** 10.1371/journal.pone.0214591

**Published:** 2019-03-27

**Authors:** Lindsey M. Christman, Lisa L. Dean, Jonathan C. Allen, Sofia Feng Godinez, Ondulla T. Toomer

**Affiliations:** 1 Department of Food Bioprocessing and Nutrition Sciences, North Carolina State University, Raleigh, NC, United States of America; 2 United States Department of Agriculture-Agricultural Research Service, Market Quality and Handling Research Unit, Raleigh, NC, United States of America; Qatar University College of Health Sciences, QATAR

## Abstract

Diabetes affects at least 285 million people globally, and this number continues to increase. Clinical complications include impaired glucose metabolism, hyperglycemia, dyslipidemia, atherosclerosis and non-alcoholic fatty liver disease. Evidence has shown that natural phenolics play a protective effect on both the development and management of type 2 diabetes. This study evaluated effects of the extract from peanut skins containing polyphenols on induced- hyperglycemia using *in vivo* and *in vitro* methods. A human hepatocellular liver carcinoma cell line (HepG2) was used to investigate the effect of the peanut skin extract on cell viability after exposure to high glucose concentrations. *In vivo*, the effect of peanut skin extract on an oral glucose tolerance was investigated in human subjects. Fifteen participants aged 21–32 underwent an oral glucose tolerance test with five treatments: 1) 50-gram glucose solution (reference); 2). 50-gram glucose solution, followed by 12 mg of vegi-capsulated maltodextrin; 3) 50-gram glucose solution, followed by 120 mg of vegi-capsulated maltodextrin-encapsulated peanut skin extract; 4). 50-gram glucose solution, followed by 28 grams of unfortified coated peanuts; 5) 50-gram glucose solution, followed by 28 grams of chili lime coated peanuts fortified with encapsulated peanut skin extract. Glucose levels were measured using a continuous monitor. Peanut skin extract was found to attenuate the decrease in cell viability in high glucose treated HepG2 cells, showing a protective effect against hyperglycemia induced cell death. No difference in the glycemic response area under the curve between any treatments using the tolerance test, but the treatment of the peanut skin extract with the glucose reference resulted in a significantly lower peak blood glucose response at 45 minutes, indicating that it was effective at reducing the glycemic response. The present study shows that the phenolic extract of peanut skins has an antidiabetic effect, further confirming their value as a functional food ingredient.

## Introduction

Diabetes mellitus, a group of metabolic diseases, is characterized by deficient insulin secretion or action and chronic hyperglycemia, and its occurrence is predicted to double by 2030 [1, 2]. Type 2 diabetes accounts for more than 90% of all diabetic cases and is the result of insulin resistance [3]. Abnormal postprandial hyperglycemia, or increased blood glucose levels, have been linked to the onset of type 2 diabetes and to the complications of diabetes [4]. Without effective management and prevention methods, the prevalence of diabetes and its corresponding complications will only continue to increase. Epidemiological evidence has shown that diets rich in foods with high total phenolic content and high antioxidant capacity may be related to lower risk of diabetes [1, 5]. As a result, the use of natural phenolic compounds has been suggested to help with the prevention and treatment of this disease [6].

Oxidative stress is caused by an imbalance between the production and elimination of reactive oxygen species. Increased oxidative stress has been linked to both the development and progression of diabetes and its associated complications [1]. Hyperglycemia, or elevated blood glucose levels, has been shown to increase reactive oxygen species production [1]. The resulting increase in oxidative stress is considered to play a major role in the complications of diabetes, such as retinopathy, nephropathy, peripheral nerve damage, and atherosclerosis [7]. Natural plant antioxidants, such as phenolic compounds, have been suggested as an effective and inexpensive way to help to prevent and manage the side effects of diabetes due to their ability to reduce oxidative stress and their effects on carbohydrate metabolism [8]. Peanut skins are rich in phenolic compounds, and their antioxidant activity has been reported in numerous studies [9, 10, 11, 12, 13]. However, there is very limited research on the effect on hyperglycemia. Tamura and others [14] found that peanut skin procyanidins inhibited the activity of alpha-amylase and decreased glucose transport in Caco-2 cells. However, the anti-diabetic effect of peanut skin phenolic compounds has not been reported in human studies.

This study investigated the effects of peanut skin extract on hyperglycemia using both *in vitro* and *in vivo* models. *In vitro*, the effect of peanut skin extract on an induced hyperglycemic-induced inflammatory responses in HepG2 cells was determined. *In vivo*, the effect of peanut skin extract on postprandial hyperglycemic responses using an oral glucose tolerance test was evaluated. Thus, this study provides insight into the biological effects of extractable compounds on peanut skins.

## Materials and methods

### Materials

Peanut skins, collected after the blanching process from Runner type peanuts were obtained from Jimbo’s Jumbos (Edenton, N.C., USA). The peanut skins were waste material from the blanching operation and were from mill run peanuts from various lots grown in Eastern North Carolina and harvested during the 2017 crop year. Maltrin M150 maltodextrin was provided by Grain Processing Corp (Mascatine, Iowa, USA). Pure pharmaceutical grade ethanol (Decan Laboratories Inc, King of Prussia, PA, USA) was used for preparation of peanut skin extract. TicaPAN 311 (Tic Gums, MD, USA) was used for pre-coating chili lime peanuts. Dulbecco’s Modified Eagle’s Medium (DMEM), Fetal Bovine Serum (FBS), trypsin- ethylenediaminetetraacetic Acid (EDTA), HEPES (4-(2-hyrdoxyethyl)-1-piperazineethanesulfonic acid, penicillin-streptomycin, glutamine, non-essential amino acid solution, N- acetyl cysteine (NAC), glucose, and trypan blue were all purchased from Gibco (Invitrogen, Carlsbad, CA, USA). The MTT cell growth assay kit was purchased from Sigma- Aldrich (Milipore Corp. Bedford, MA, USA). The Alanine Transaminase assay kit (ALT) was purchased from Biovision (Minneapolis, MN, USA). Standard 50-gram glucose solutions for glucose load were obtained from Azer Scientific (Morgantown, PA, USA). Sterilize lancet, glucose meter equipment and strips for glucose meter were obtained from One Touch (Lifespan Inc., Wayne, PA, USA).

### Preparation of peanut skin extract and coated peanuts

The extract from peanut skins was prepared according to the protocol by Constanza and others [15]. Briefly, peanut skins were ground using a Blixer-3 food processor (Robot Coupe, Jackson, MS, USA) and extracted with 70% pure ethanol and deionized water solution in a 1:5 skins to solvent ratio. The solution was stirred for 20 minutes at a speed of 2.8 using a Wheaton overhead stirrer (Wheaton Industries, Inc. Milville, NJ, USA). The soluble portion of the extract was separated from the insoluble fiber by vacuum filtration using Whatman #40 filter paper (GE Healthcare, Marlborough, Mass, USA). The ethanol was evaporated from the soluble extract using a Büchi Rotovap (Büchi Labortechnik, Switzerland) at 60° C. The extraction was performed under low actinic lighting to minimize degradation of polyphenols under light.

The aqueous extract was split in half. For the oral glucose tolerance test, the aqueous extract was mixed with 10.5% (w/w) maltodextrin and was allowed to hydrate for a minimum of 24 hours. For the cell study, the peanut skin extract (PSE) was not mixed with maltodextrin. Both extracts were fed into a Buchi Model B-290 bench scale spray dryer (Buchi Labortechnik, Switzerland). The settings included an inlet temperature of 175° °C, an outlet temperature of 90° °C, a solution feed pump of 30%, the nitrogen flow rate of 35 psi, and the aspirator set at 100%. The dried powders were collected for the study as described below. Total phenolic content, DPPH radical scavenging activity and β-carotene bleaching activity of the resulting PSE powders were determined and reported elsewhere [16]

Peanuts were blanched using a whole nut blancher (Ashton Food Machinery Co. Inc., Newark, NJ) prior to processing. The peanuts were oil roasted using a P-H-T Fryer model 500 (Henry Penny Corporation, Eaton, Ohio) filled with 8 gallons of peanut oil (Stratas Food, Memphis TN). After the roasting, the peanuts were spread on a wire mesh screen with a cooling fan above to cool to ambient temperature. Roasted peanuts were coated in 33% Tic Precoat 311 solution and 67% chili lime seasoning blend (Trader Joe’s, Monrovia, CA). Maltodextrin encapsulated PSE was added to chili lime seasoning at 16.6% (w/w), based on a threshold test [16]. The peanuts were placed in a stainless steel panner rotating at 30 rpm. The Tic Precoat 311 solution was first applied to the peanuts, followed by the chili lime seasoning. Peanuts were rotated in the panner until evenly coated.

### Cell culture

The human hepatocellular liver carcinoma cell line (HepG2 cells), obtained from American Type Culture Collection (ATCC, Manassas, VA, USA), were cultured in complete media containing, Dulbecco’s modified Eagle’s medium (DMEM) supplemented with 10% heat inactivated fetal bovine serum (FBS) and 2% of penicillin streptomycin, glutamine, 4-(2-hyrdoxyethyl)-1-piperazineethanesulfonic acid (HEPES), pyruvate, and non-essential amino acids under 5% CO_2_ atmosphere at 37°C. When the cells reached 80–90% confluence, cells were sub-cultured using trypsin-ethylenediaminetetraacetic acid (EDTA) to detach from the flask. Cells were plated 48 hours prior to treatment.

### Treatment of cells

The toxicity of PSE on HepG2 cells was first screened to determine the correct dosage to use for further testing. HepG2 cells were incubated for 24 hours at 37°C and 5% CO_2_ with media containing doses of 0, 0.5%, 1%, 2.5%, 5%, and 10% PSE. Following the 24-hour incubation, cells were tested for cell proliferation, viability and ALT enzyme activity. Various concentrations of glucose were screened to determine the correct dosage to induce a condition of oxidative stress in HepG2 cells. HepG2 cells were treated with media containing 0, 20, 40, 60, 80, 100, 130, and 160 mM glucose for 24 hours at 37°C and 5% CO2. Following the 24-hour incubation, cells were analyzed for cell proliferation, viability and ALT enzyme activity.

Based on the screening results, cells were divided into 5 experimental groups: i. Control (Untreated); ii. Cells treated with glucose; iii. Cells treated with PSE; vi. Cells were treated with PSE and glucose; v. Cells treated with N-acetyl cysteine (NAC) (5 mM); iv. Cells treated with NAC and glucose (positive control). Cells were incubated with treatments for 24 hours at 37°C and 5% CO2. Following the 24-hour incubation, cells were analyzed for cell proliferation, viability and ALT enzyme activity.

### MTT cell growth assay

The MTT (3(4,5-dimethylthiazol-2-yl)-2,5-diphenyltetrazolium bromide) assay was used to determine the cell proliferation. After the 24- hour incubation, cells were plated in 96-well plates (4x10^5^ cell/well) and measured using the MTT Cell Growth Assay Kit according to manufacturer’s instructions. Briefly, 0.01 mL MTT reagent was added to each well. The plate was then incubated for 4 hours at 37°C to allow for cleavage of MTT to occur. Each well was then treated with 0.1 mL isopropanol with 0.04 N HCl and the absorbance was read at 570 nm using an Epoch 25 Microplate Spectrophotometer (Biotech, Winooski VT, USA). The values were corrected by subtracting out the background absorbance of media containing peanut skin extract with no cells. Cell viability was expressed as a percentage of proliferation of the control based on Eq 1. The viability of the control was set at 100%.

$$\frac{\left(Sample\;Absorbance\right)}{\left(Control\;Absorbance\right)}\;X\;100\%=\%\;Relative\;Cell\;Viability$$ Eq 1

### Trypan blue exclusion method

Cell viability was measured using the trypan blue exclusion method. Briefly, the cell suspension was treated with trypan blue stock solution in a 1:1 ratio. The mixture was placed in a TC20 Automated Cell Counter (BioRad, Hercules, CA, USA) to detect the cells that had absorbed the dye in order to calculate the percentage of viable cells. Data was expressed as percent viability relative to the control.

### ALT enzyme activity assay

The Alanine Aminotransferase (ALT) activity was measured for treated cells using Biovisions ALT activity colorimetric assay (Biovision, Minneapolis, MN, USA). All manufacturers’ instructions were followed.

### Oral glucose tolerance test

#### Subjects

Fifteen healthy subjects (7 males and 8 females) with an age range of 23–32 were recruited from the staff and student population of North Carolina State University, Raleigh, NC to participate. All participants were frequent consumers of peanut products. Exclusion criteria for participants in this study included: BMI> 35 kg/m^2^, diagnosis of type 1 or 2 diabetes, anemia, a fasting glucose >125 mg/dL, use of medication that affect glucose metabolism, renal, liver, pancreatic, or cardiovascular disease, uncontrolled hypertension, disorders of the esophageal or in gastrointestinal motility, hypo- or hyperthyroidism, allergic to peanuts, and pregnancy. The study was approved by the North Carolina State University institutional review board and all subjects gave written consent after a full explanation of the study procedures.

#### Composition of experimental drinks

Each participant completed five treatments. The five experimental treatments included: 1) 50 g glucose solution (reference); 2). 50-gram glucose solution, followed by 12 mg of vegi-capsulated MD (placebo); 3) 50-gram glucose solution, followed by 120 mg of vegi-capsulated MD-encapsulated PSE (Treatment); 4). 50-gram glucose solution, followed by 28 grams (1 serving) of unfortified coated peanuts; 5) 50-gram glucose solution, followed by 28 grams of chili lime coated peanuts fortified with PSE. The amount of peanut skin extract used had equal antioxidant activity to blueberries measured by the DPPH reducing assay [15]. The antioxidant activity of PSE was previously determined by Christman [16]. The inclusion level of PSE in the chili lime flavor coating was chosen based on the detection threshold for PSE in the coating previously determined by Christman [16]. A randomized crossover design was used and each subject received each of the five treatments on different days in randomized order. This was repeated three times for each subject with a week washout period between each replication.

#### Experimental design

Subjects were instructed to refrain from eating and any non-habitual exercise at least 10 hours before treatments. They were also asked to refrain from alcohol for the entirety of the test. Otherwise, they were told to maintain their usual dietary intake, and to keep a record of their food intake, number of steps, and physical activity level.

A MiniMed iPro 2 professional continuous glucose monitor (CGM, Medtronic) was inserted into the lower abdomen of all subjects one day before they started treatments. The CGM was used to continuously measure the participant’s interstitial glucose levels and provide a glycemic response profile for the study. It operates for 72 hours and records a glucose measurement every 5 minutes. After the five days of treatments, CGMS were removed and the data was downloaded and analyzed using CGMS Software version 3.0.

On each day of the study, the participants were instructed to take a fasting blood glucose measurement before consuming the treatment (time zero) by finger prick using a blood glucose meter (OneTouch Verio, IQ) in order to calibrate the CGM. They were then instructed to take one of their five treatments within ten minutes. For the next 2.5 hours, they were instructed to refrain from eating and any non-habitual exercise. A second blood glucose measurement was taken 2.5 hours after treatment by finger prick. The fasting blood glucose level determined before the treatment were used as a baseline and the change in glucose levels for the 2.5 hours after each treatment were plotted.

The glycemic response for each treatment was estimated by calculating the total incremental area under the curve (AUC) for the treatment using the trapezoidal model [17]. Peak heights on the glycemic response curve were the incremental rises in blood glucose level above the baseline fasting glucose level. The glycemic profile was calculated by dividing the duration the glucose level was above the fasting glucose level by the incremental glucose peak [18]. When the glucose remained above fasting the entire 150 minutes after treatment, the duration value was set to 150 minutes.

### Statistical analysis

Experiments were done in triplicate and data are expressed as mean ± SEM. Statistical analysis was performed using SAS version 9.4 (SAS Inst., Cary, NC). Cell viability measured by both MTT assay and trypan blue exclusion method were expressed as percent viability relative to the control. Differences in cell viability and ALT enzymes activity between treatments was determined by one-way ANOVA followed by Tukey post hoc test. AUC, peak height, and glycemic profile were analyzed using a one-way ANOVA with repeated measures and were compared by Tukey post hoc test. P values <0.05 were considered significant.

## Results

### Toxicity of peanut skins

**Fig 1:**
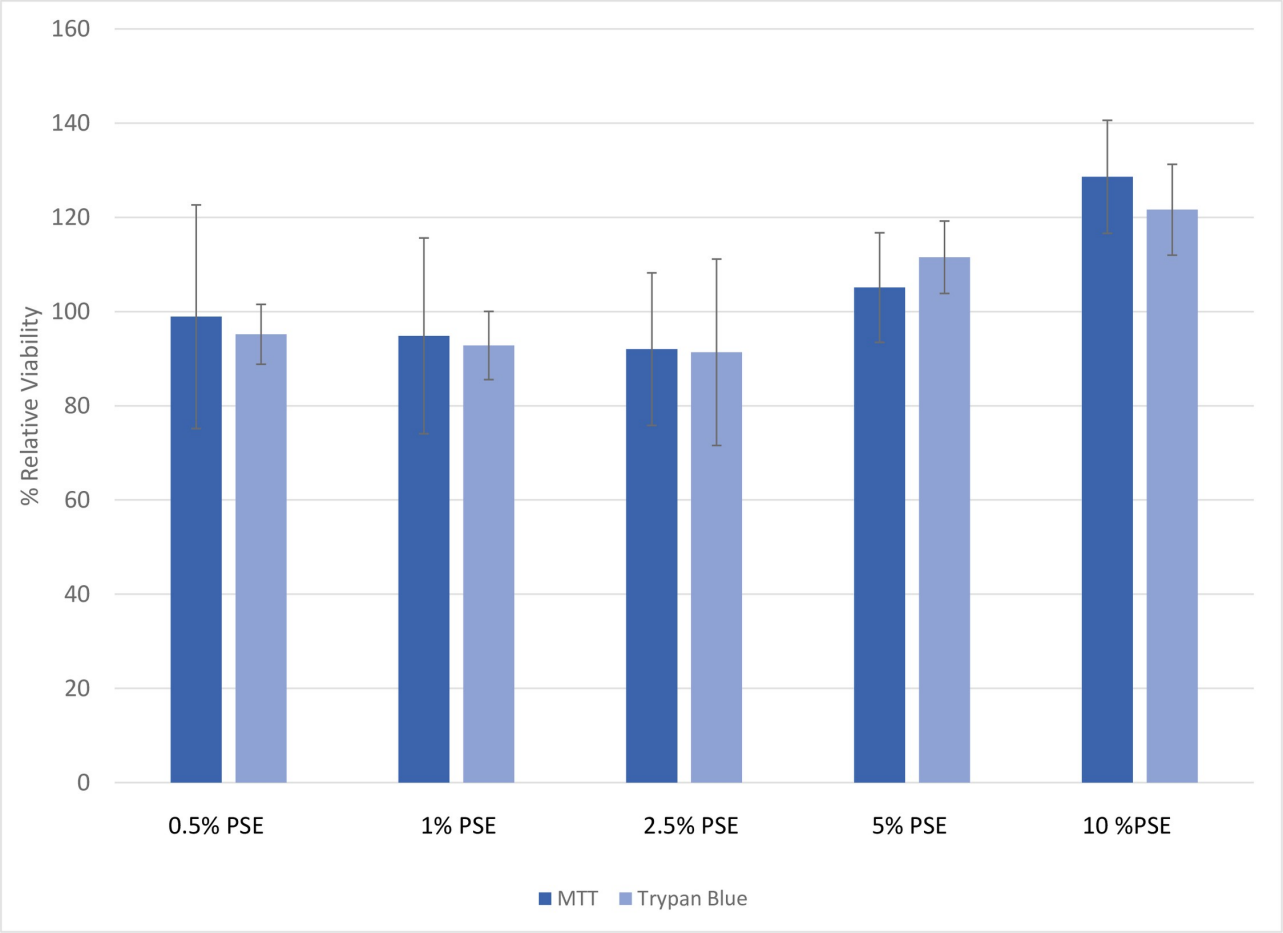
Effects of varying concentrations of PSE on HepG2 cells viability measured by (A) MTT cells growth assay and (B) trypan blue exclusion method. HepG2 cells were exposed to varying concentrations of peanut skin extract (0–10%) for 24 hours and their viability was determined. The viability of the control was assumed to be 100% and the data are expressed as viability relative to the control. Each treatment was done in triplicate for both methods and the data is expressed as mean ± SEM. *Indicates statistically significant difference at P<0.05.

HepG2 cells were used as an *in vitro* model to investigate the biological effects of PSE on hyperglycemic-induced hepatic responses due to the role of the liver in glucose metabolism and regulation [19]. To identify an efficacious and non-lethal experimental dose of PSE, HepG2 cells were exposed media supplemented with a range of PSE concentrations (0–10%) for 24 hours. The concentrations of PSE tested in this study did not result in significant changes in cell viability (Fig 1). This indicates that exposure to these concentrations of PSE for 24 hours was non-lethal to HepG2 cells.

**Fig 2:**
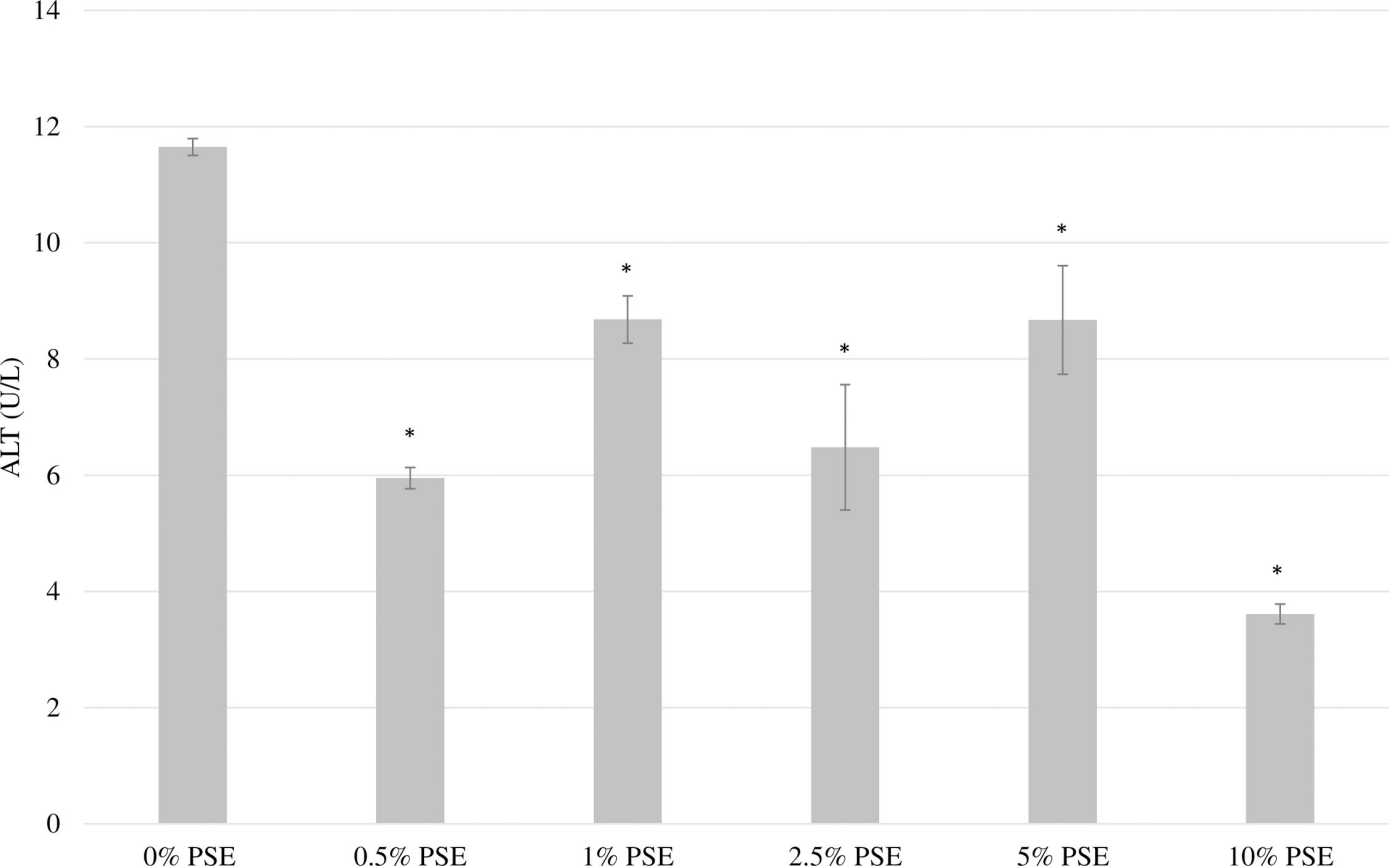
Effect of varying concentrations of peanut skin extract on ALT leakage from HepG2 cells. HepG2 cells were exposed to varying concentration of peanut skin extract for 24 hours and the level of transaminase enzyme were measured. Each treatment was done in triplicate and data are expressed as mean ± SEM. *Indicates statistically significant difference at P<0.05.

Additionally, there was no significant increase in ALT activity when cells were treated with all experimental levels of peanut skin extracts. As shown in Fig 2, treatment with PSE was found to cause a significant decrease in ALT activity, suggesting the PSE may help to protect the cells against any natural stresses that were not controlled for. ALT, an enzyme that catalyzes the transfer of amino groups to form the hepatic metabolite, oxaloacetate, is mainly found in the liver [20]. An increase in the measured activity in the cell medium demonstrates a release of ALT from the liver due to a cellular dysfunction [20]. A reduction in ALT activity within the cell medium implies improved cellular liver function and enhanced cellular protection of HepG2 cells.

### Toxicity of glucose

**Fig 3:**
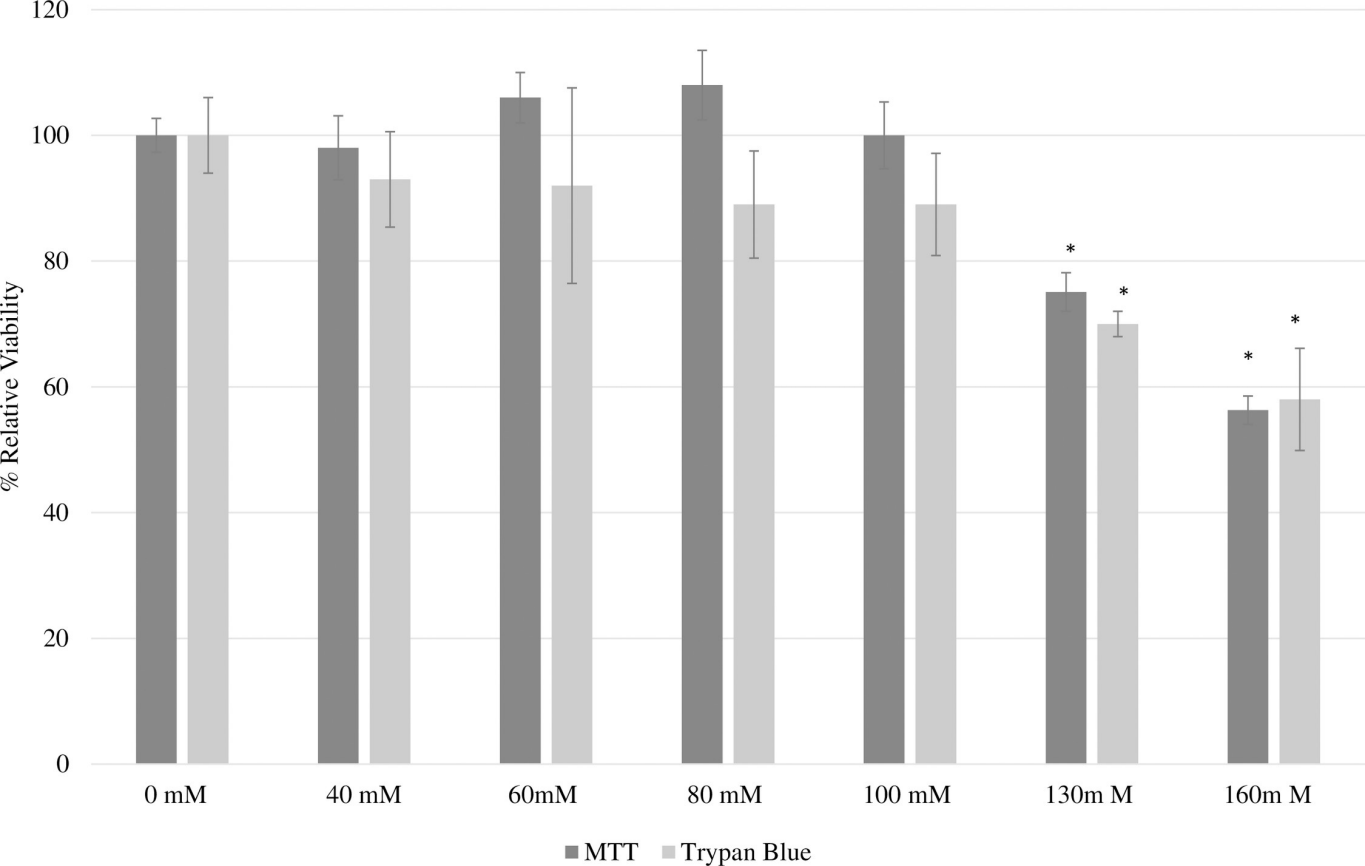
Effects of varying concentration of glucose on HepG2 cells viability measured by (A) MTT cells growth assay and (B) trypan blue exclusion method. HepG2 cells were exposed to complete DMEM media supplemented with varying concentrations of glucose (0–160 mM) for 24 hours and their viability was determined. The viability of the control was assumed to be 100% and the data are expressed as viability relative to the control. Each concentration was done in triplicate for both methods and the data is expressed as mean ± SEM. *Indicates statistically significant difference at P<0.05.

**Fig 4:**
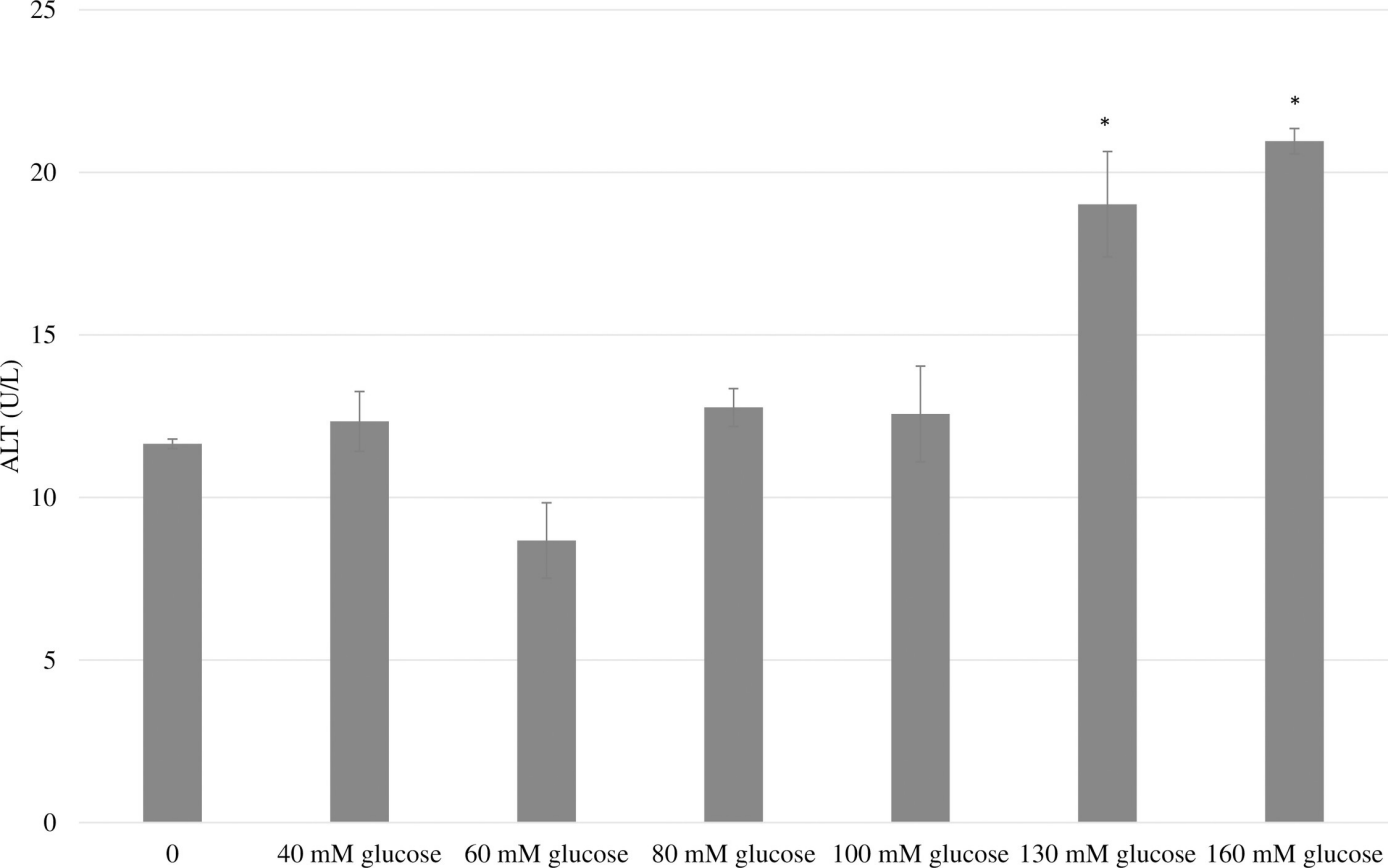
Effect of varying concentrations of glucose on ALT leakage from HepG2 cells. HepG2 cells were exposed to complete DMEM media containing varying concentrations of glucose for 24 hours and the level of transaminase enzyme were measured. Each treatment was done in triplicate and data are expressed as mean ± SEM. *Indicates statistically significant difference at P<0.05.

HepG2 cells were exposed to media with various concentrations of glucose (0-160mM) for 24 hours to identify dose-responsive hyperglycemic-induced responses. As shown in Fig 3, incubation of the cells with glucose at a concentration of 130 and 160 mM for 24 hours was found to significantly lower the cell viability when measured by both the MTT cell growth assay and trypan blue exclusion method. This concentration is high in comparison to others studies that have found that exposure of HepG2 cells to 50 mM of glucose for 24 hours was sufficient to significantly reduce cell viability [8, 19]. However, due to the results of this study, a concentration of 160 mM was utilized in additional experimentation. As shown in Fig 4, exposure to 160 mM of glucose also resulted in a significant increase in ALT activity.

### Effect of PSE on high glucose treated cells

**Fig 5:**
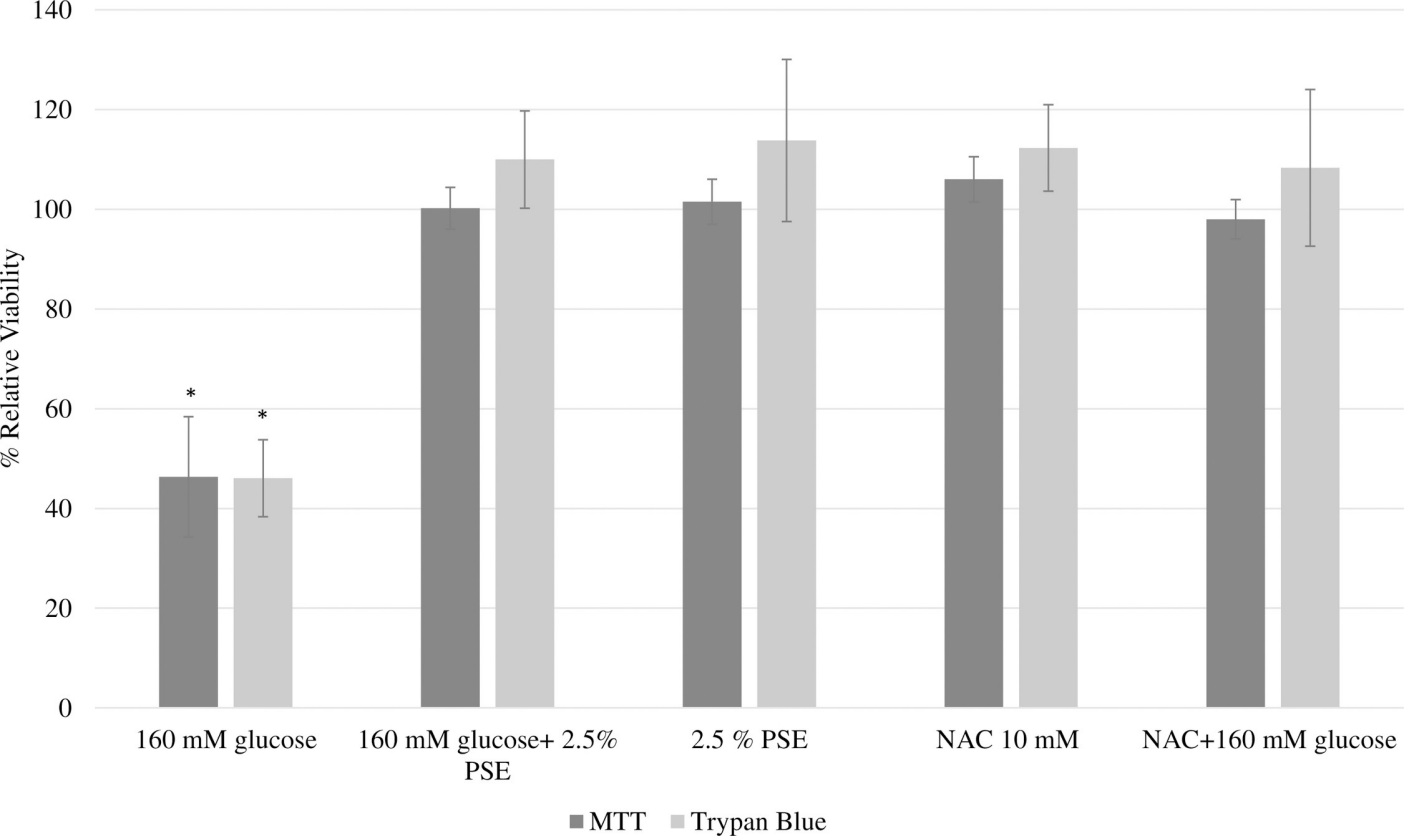
Effect of PSE on high glucose induced cytotoxicity in HepG2 cells viability measured by (A) MTT cell growth assay and (B) trypan blue exclusion method. HepG2 cells were exposed to either glucose, peanut skin extract (PSE), glucose and peanut skin extract (PSE), n-acteyl cysteine, or n-acetyl cysteine and glucose for 24 hours. N-aceytl cysteine is an antioxidant and used as a positive control. The viability of the control was assumed to be 100% and the data are expressed as viability relative to the control. Each treatment was done in triplicate and data are expressed as viability relative to the control and as mean ± SEM. *Indicates statistically significant difference at P<0.05.

**Fig 6:**
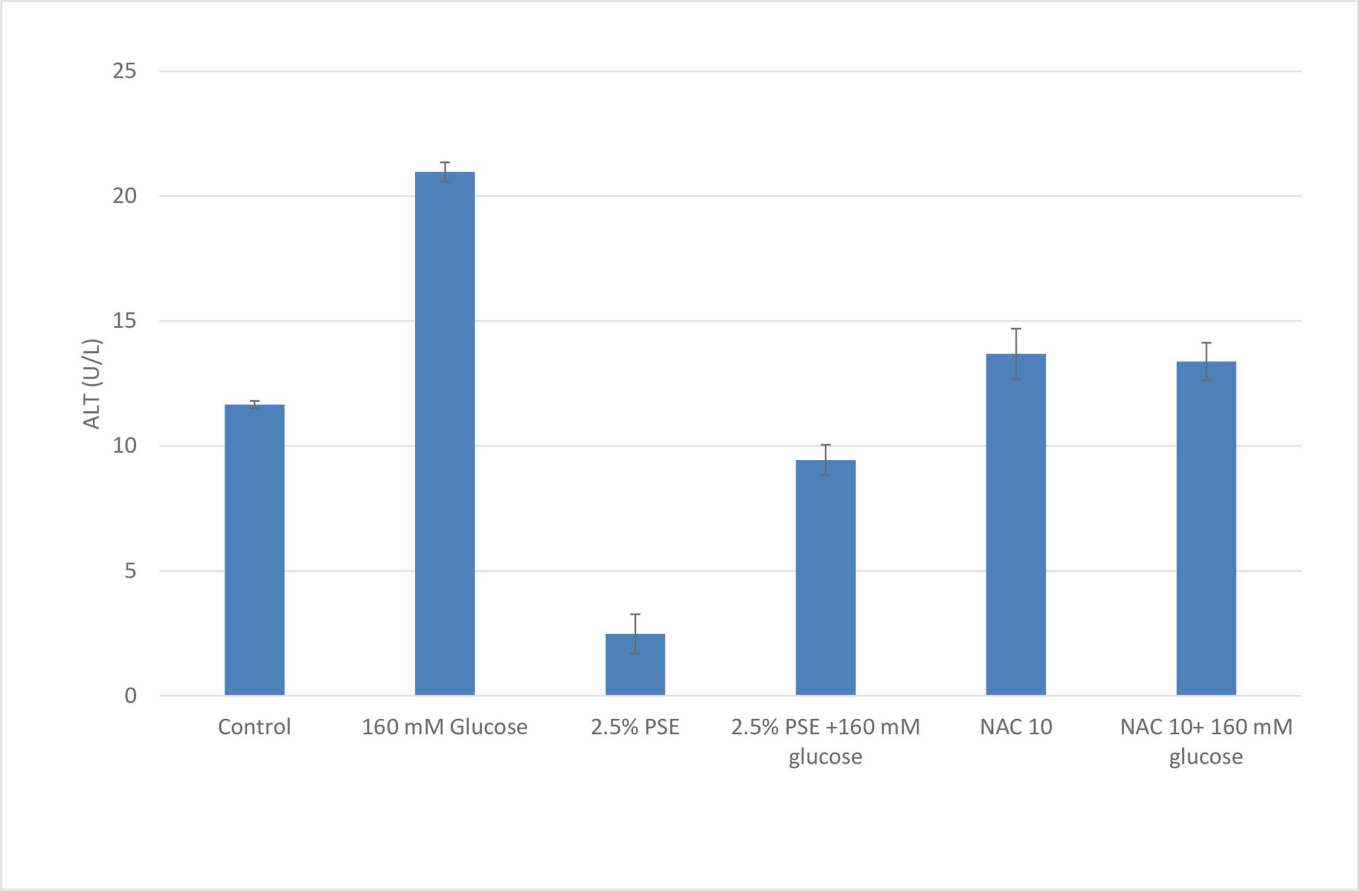
Protective effect of PSE on ALT leakage from HepG2 cells. HepG2 cells were exposed to either glucose, peanut skin extract (PSE), glucose and peanut skin extract (PSE), n-acetyl cysteine (NAC) or n-acetyl cysteine and glucose for 24 hours. N-acetyl cysteine is an antioxidant and used as a positive control. Each treatment was done in triplicate and data are expressed as mean ± SEM. *Indicates statistically significant difference at P<0.05.

Based on the results of the toxicity screening studies, HepG2 cells exposed to media containing both 2.5% PSE and 160 mM glucose for 24 hours, demonstrated that PSE at the 2.5% dose had a protective effect on cells exposed to 160 mM of glucose (Fig 5). Exposure of cells to 160 mM of glucose caused the relative cell viability reduction of 46.3% and 58.4%, when measured by MTT and trypan blue exclusion, respectively. However, co-treatment of the cells with 2.5% PSE and 160 mM glucose brought the relative cell viability to above 100% when measured by both methods, suggesting that PSE had a protective effect against hyperglycemic-induced cellular dysfunction. Similar results were found for the co-treatment of cells with the positive control, NAC (antioxidant), and 160 mM glucose. Treatment with PSE also attenuated the increase in ALT activity induced by high glucose concentrations, further suggesting the protective effect of PSE (Fig 6).

### Oral glucose tolerance test

A total of 15 people (male = 7 and female = 8) aged 21–32 completed this intervention study to investigate the effects of PSE on the glycemic response to consuming a 50-gram glucose solution. An oral glucose tolerance test was used as this is the common method for assessment of the postprandial glycemic response [21]. Although consuming a pure glucose solution does not cause the same metabolic response as a mixed meal, it has been shown that the level of glycaemia after drinking a glucose load of 50 or 75 grams is closely related to the level of glycaemia after a standardized meal [22].

**Fig 7:**
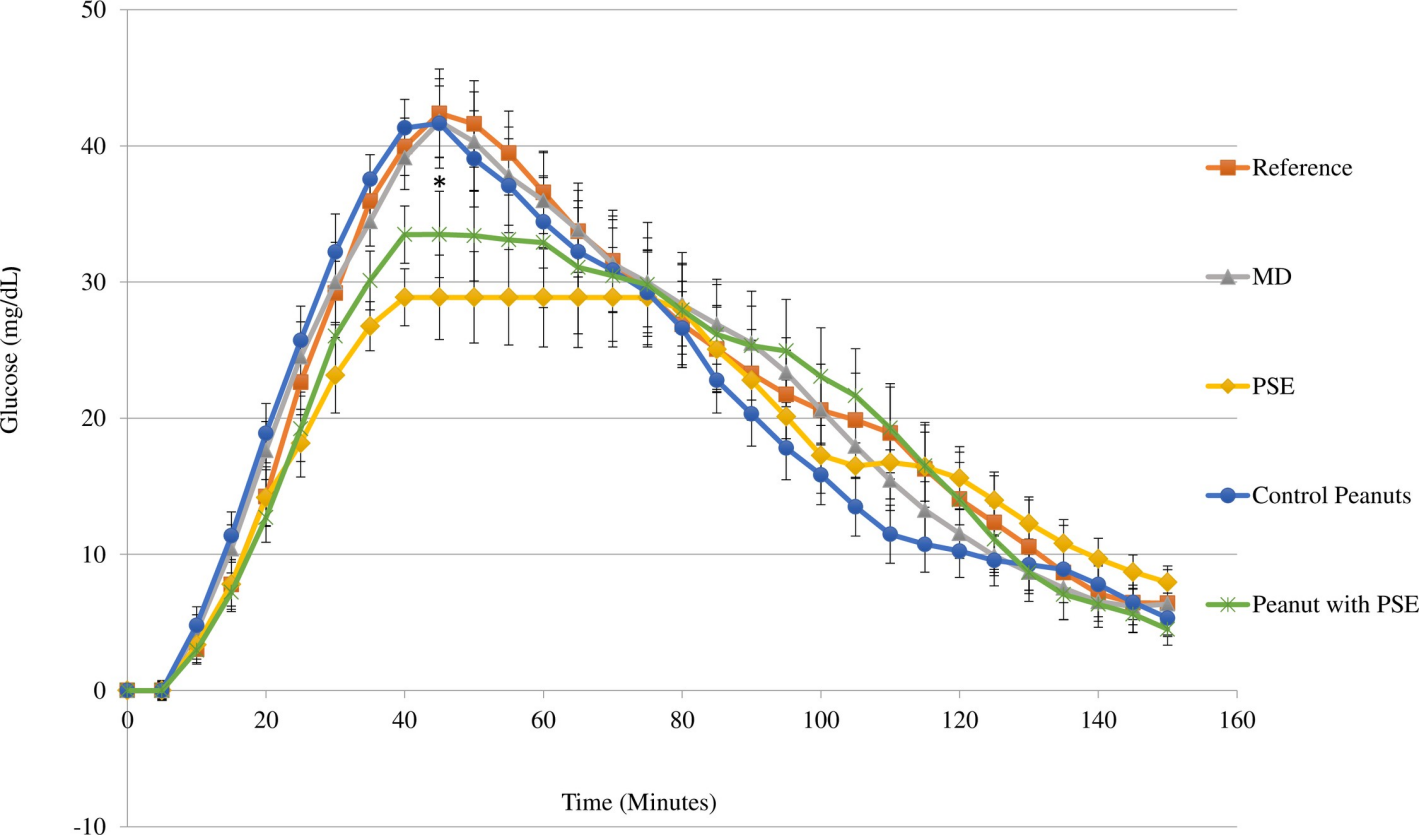
Postprandial change in glucose level from baseline (mean ± SEM) during an oral glucose tolerance test. The fasting glucose level was used a baseline and the change in glucose level is plotted over time for 2.5 hours of the oral glucose tolerance test. Treatments for the oral glucose tolerance test include 50 gram glucose solution either alone (□), with capsule filled with maltodextrin (MD) (▲),with capsule filled with peanut skin extract (PSE) (♦), with PSE- fortified peanuts(●), or with non-fortified peanuts(*) in 15 healthy subjects. Each subject consumed each treatment in triplicate. *Indicates statistically significant difference between treatments (P<0.05).

Thirteen people had data for the complete three-week test period, while two people only had data for two weeks due to inaccuracies in the CGM. Inaccuracies in the CGM data are a result of the calibration of the interstitial fluid glucose with the blood glucose levels [23]. The mean change in glucose concentrations from fasting level were plotted as a function of time for 150 minutes after each treatment (Fig 7). There was no significant difference when comparing the AUC for the different treatments after 150, 120 and 60 minutes (Table 1). However, the consumption of the PSE capsule and PSE fortified coated peanuts with the glucose load caused a significant decrease in the peak glucose response at 45 min. High postprandial glucose spikes are associated with increased risk of developing chronic disease, suggesting that this decrease in the glucose peak at 30–45 minutes may be more important than the overall AUC [24, 25].

**Table 1:** Blood glucose responses in 15 healthy individuals after 5 treatments in oral glucose tolerance test. The change in glucose level from fasting level was plotted against the time for 2.5 hours in glycemic response curve. Glucose levels were measured using a continuous glucose monitor. The area under the curve (AUC), glycemic profile, and glycemic incremental peak were determined from the glycemic response curve.

Treatment	AUC (0–30)	AUC (0–60)	AUC (0–120)	AUC (0–150)	GP Min/mg/dL	Glycemic incremental peak (mg/dL)
50 gram glucose solution	742.4± 74.8b	1743.8± 292.1a	3107.5± 342.0a	3144.5± 344.6a	3.06±1.47a	42.7± 2.8b
Maltodextrin capsule	750.5± 76.8b	2033.5± 298.6a	2944.5± 328.4a	3139.9 ± 350.4a	3.07±1.51a	42.3± 2.8
PSE capsule	466.2± 75.8a	1880.4± 299.1a	2971.9± 330.9a	2984.14± 347.4a	8.99±1.48b	27.8± 2.8a
Unfortified chili lime coated peanuts	802.0± 75.8b	2073.7± 288.7a	3207.4± 323.7a	3387.1 ± 342.9a	3.15± 1.46a	43.4±2.7b
PSE fortified chili lime coated peanuts	687.2± 73.9b	1812.7± 291.5a	3054.1± 321.7a	3144.9± 339.4a	5.94± 1.47ab	33.5± 2.7a

Each treatment was done in triplicate for each individual. Values are means ± SEM. Products in the same column not sharing the same letter were significantly different p< 0.05.

The glycemic profile is a variable used to quantify the change in the shape of the glucose curve [18]. It is defined as the duration of the postprandial blood glucose level above the baseline fasting level divided by the blood glucose peak. The PSE capsule treatment was found to significantly increase the glycemic profile compared to the control (Table 1). A higher glycemic profile is suggested to indicate facilitated glycemic regulation, further demonstrating the effect of PSE on glycemic response.

## Discussion

Peanut skins, a low value waste product of the peanut industry, are rich in phenolic compounds. Their antioxidant activity has been reported in various studies through *in vitro* chemical assays [9, 10,12,13, 15, 26]. However, few studies have examined their biological activity [11, 14, 27, 28]. The present study investigates the biological activity of phenolic extracts from peanut skins through studying their effect on hyperglycemic-induced responses using *in vivo* and *in vitro* methods.

The present study demonstrates that PSE had a protective effect on HepG2 cells exposed to lethal glucose concentrations. This effect may be due to the ability of PSE to act as antioxidant and reduce the oxidative stress induced by hyperglycemia. Several studies have shown that the toxicity of high glucose concentrations on HepG2 cells has been associated with an increase in oxidative stress [19, 29]. Chandrasekaran and others [19] found exposure of HepG2 cells to high glucose concentrations caused an increase in oxidative stress markers such as reactive oxygen species (ROS). There was also a 2-fold increase in the measured lipid peroxidation and protein carbonyls, a modification of proteins that occurs due to oxidation, in cells exposed to high glucose. Similarly, Cordero-Herra and others [29] found that exposure of HepG2 to high levels of glucose resulted in an increased production of ROS and increased activity in innate antioxidant enzymes.

The observed protective effect of PSE may be due to their ability to act as antioxidants and reduce this oxidative stress. The positive control, NAC, is an antioxidant that leads to the generation of GSH, an innate antioxidant enzyme in the body. As shown in Fig 5, NAC and PSE were found to have a similar effect on glucose treated cells. Chandrasekaran and others [19] found that NAC was able to attenuate the decrease in cell viability in HepG2 cells exposed to high glucose levels by lowering the reactive oxygen species level. The similar results found between PSE and NAC suggest that they may have a similar mechanism of action.

Numerous studies have found similar results on the effect of natural phenolic compounds on high glucose induced oxidative stress [8,29,30]. For example, Kapoor and Kakkar [30] found that morin, a natural flavonoid found in many fruits, herbs, and wine, was able reduce ROS levels in high glucose treated rat hepatocyte cells, resulting in the inhibition of the release of apoptotic proteins from the mitochondria. Similar results were found for the phenolic extract of cocoa [29]. In addition, it was found that this decrease in ROS production inhibit the activation of key signaling proteins. Mitogen-activated protein kinases (MAPks), such as extracellular signal-regulated kinase (ERK), c-Jun-N- terminal kinase (JNK), and p-38, are activated by oxidative stress, and are responsible for altering the expression of genes involved in high glucose induced apoptosis, inflammation, and oxidative stress [24]. The co-treatment of high glucose levels and the cocoa phenolic extract was found to prevent the activation of these kinases in HepG2 cells, suggesting this is the mechanism for how phenolics can alleviate the damages of oxidative stress in the hyperglycemic state [29].

In the present study, we also investigated the effect of PSE on the glycemic response to a 50-gram glucose solution. PSE treatments were found to decrease the peak plasma glucose level, suggesting that the phenolic compounds may be delaying the absorption of glucose. Consistent with these findings, peanut skin phenolic fractions, including (+)- catechin, procyanidin A1, and epicatechin-(4β→ 6)-epicatechin-(2β→ 0—>7, 4β→ 8)-catechin (EEC), have been found to decrease glucose uptake in Caco-2 cells [14]. Although the mechanism was not investigated in this study, these phenolic compounds have been found to inhibit glucose transporters in the intestine. The two major glucose transporters in the intestine include the sodium-dependent glucose co-transporter 1 (SGLT1) and sodium-independent transport glucose transporter (GLUT2). Tea polyphenols such as epicatechin gallate (ECg) and epigallocatechin gallate (EGCg) have been shown to inhibit SGLT1 through competitively binding [31]. Phenolic compounds extracted from strawberries and apples, similar to those extracted from peanut skins, were also found to inhibit SGLT1 and GLUT2 by binding and interacting with the transporters [32]. This inhibition of glucose transport would result in a lower postprandial glycemic response.

Phenolic compounds found in peanut skins have also been found to activate signaling pathways involved in glucose transport. Glucose transporter 4 (GLUT4), located within the intracellular compartment of adipose tissue, and skeletal and cardiac muscles [33], is translocated to the plasma membrane in response to insulin. This allows for a large amount of glucose to be brought into the cell, helping to reduce blood glucose levels and maintain glycemic homeostasis in the body. GLUT4 translocation is regulated by 5’adenosine monophosphate-activated protein kinase (AMPK) and phosphoinositide-3- kinase (P13K) [34]. Treatment of cultured L6 myotube cells with resveratrol has been shown to cause the translocation of GLUT4 by activating AMPK-dependent signaling pathways [35]. Resveratrol is the major stilbene that is found in peanut skins [36]. Similarly, the procyanidin extract from cacao liquor was found to improve high-fat diet-induced hyperglycemia in mice by activating AMPK and causing translocation of GLUT4 in skeletal muscle, and white and brown adipose tissue [33]. This procyanidin extract was rich in phenolic compounds found in peanut skins such as, monomeric epicatechin and catechin, and oligomeric procyanidins [37].

The co-treatment of peanuts and the glucose solution resulted in a slight increase in the blood glucose peak. Previous studies have shown that peanut consumption can lead to a reduced glycemic response as a result of the fat and protein content [38]. This was suggested to be a result of the fat and protein content causing an increase in gastric emptying and increase in insulin secretion. The contrasting results of this study may be due to the difference in form of the peanut. The previous study found that ground peanuts were able to significantly lower the glycemic response in comparison to whole peanuts because the amount of fat released and absorbed in the digestive system is dependent on the integrity of the cell wall [39]. The grinding process required to grind the peanuts was thought to cause more destruction to the cell wall than is possible with mastication alone. This explains the lack of effectiveness of the un-fortified peanuts on the glycemic response. The treatment size of peanuts in this study may have been too small. The treatment size for this study was chosen as one serving because it was a realistic amount for a person to consume. However, Josse and others [40] found that 60 grams of almonds were needed to decrease the glycemic response to white bread. The single serving size used for this study may not have been sufficient to cause a response.

## Conclusion

The present study demonstrated the protective effects of extracts from peanut skins containing phenolic compounds on hyperglycemic responses both *in vitro* and *in vivo*. PSE was found to protect HepG2 cells from toxicity of high levels of glucose. PSE was also able to lower the postprandial glucose spike after consuming a glucose load. A larger study on the effects of PSE on the glycemic response is needed to confirm these results. In addition, future studies should investigate the effect of PSE on glucose transporters to better identify how glucose transport is altered with PSE treatment. This research helps to elucidate the biological effects of peanut skin phenolic *in vivo* and *in vitro*, further confirming the potential as a functional food ingredient.

## Abbreviations

PSE: peanut skin extract
ROS: reactive oxygen species
DMEM: Dulbecco’s modified eagle’s medium
FBS: fetal bovine serum
EDTA: ethylenediaminetetraacetic acid
NAC: N-acetyl cysteine
MTT: (3-(4,5-Dimethylthiazol-2-yl)-2,5- Diphenyltetrazolium Bromide
ALT: alanine transaminase
MD: maltodextrin
DPPH: 2,2-diphenyl-1-picrylhydrazyl
CGM: continuous glucose monitor
AUC: area under the curve
GR: glutathione reductase
GPx: glutathione peroxidase
GST: glutathione S-transferase
CAT: catalase
MAPKs: mitogen-activated kinases
ERK: extracellular signal-regulated kinase
JNK: c-Jun-N-terminal kinase
EEC: epicatechin- (4β→6)-epicatechin-(2β→0→7, 4β→8) catechin
SGLT: sodium glucose cotransporter
GLUT 2: glucose transporter 2
GLUT4: glucose transporter 4
ECg: epicatechin gallate
EGCg: epigallocatechin gallate
GIP: gastric inhibitory polypeptide
GLP-1: glucagon-like peptide 1
AMPK: 5’adenosine monophosphate-activated protein kinase
PI3K: phosphoinositide 3-kinase
